# Differential responses of *Ceratitis capitata* to infection by the entomopathogenic fungus *Purpureocillium lilacinum*

**DOI:** 10.1371/journal.pone.0286108

**Published:** 2023-09-28

**Authors:** Wafa Djobbi, Meriem Msaad Guerfali, Agnès Vallier, Kamel Charaabi, Hubert Charles, Justin Maire, Nicolas Parisot, Haytham Hamden, Salma Fadhl, Abdelaziz Heddi, Ameur Cherif

**Affiliations:** 1 Laboratory of Biotechnology and Nuclear Technologies, LR16CNSTN01, National Center of Nuclear Sciences and Technologies, Ariana, Tunis, Tunisia; 2 Univ Lyon, INRAE, INSA-Lyon, BF2i, UMR 203, Villeurbanne, France; 3 University of Manouba, LR11-ES31 Biotechnology and Bio-Geo Resources Valorization, Higher Institute for Biotechnology, Sidi Thabet Biotechpole, Sidi Thabet, Ariana, Tunisia; University of Carthage, TUNISIA

## Abstract

The medfly *Ceratitis capitata* is one of the most damaging fruit pests with quarantine significance due to its extremely wide host range. The use of entomopathogenic fungi constitutes a promising approach with potential applications in integrated pest management. Furthermore, developing insect control methods can involve the use of fungal machinery to cause metabolic disruption, which may increase its effectiveness by impairing insect development. Insect species, including *C*. *capitata*, relies on reproduction potential, nutrient reserves, metabolic activities, and immune response for survival. Accordingly, the purpose of this study was to investigate the impacts of the entomopathogenic fungus *Purpureocillium lilacinum* on *C*. *capitata* pre-mortality. The medfly V8 strain was subjected to laboratory bioassays, which consisted on determining the virulence of *P*. *lilacinum* on the medfly. *Purpureocillium lilacinum* was applied on abdominal topical of 5-day-old males and females. Following the fungal inoculation, we have confirmed (i) a significant increase in tissue sugar content, (ii) a significant decrease in carbohydrase activities, digestive glycosyl hydrolase, and proteinase activities in whole midguts of treated flies, (iii) the antimicrobial peptides (AMPs) genes expression profile was significantly influenced by fly gender, fly status (virgin, mature, and mated), and time after infection, but infection itself had no discernible impact on the AMPs for the genes that were examined. This study provides the first insight into how *P*. *lilacinum* could affect *C*. *capitata* physiological mechanisms and provides the foundation for considering *P*. *lilacinum* as a novel, promising biocontrol agent.

## Introduction

The Mediterranean fruit fly (medfly), *Ceratitis capitata* (Wiedemann) (Diptera: Tephritidae), is widely distributed and represents an invasive agricultural pest that affects a wide range of host fruits [1–3]. Chemical treatments are an effective tool in Tunisia for medfly management. However, repeated pesticide use causes a specific selection pressure that generates pesticide resistance [4]. Microorganisms like bacteria, fungi, and viruses have been used to develop environmentally friendly medfly control strategies. Recently, the use of entomopathogenic fungi has emerged as a promising strategy for agricultural insect pest control. The use of entomopathogenic fungi as biocontrol agents is critical for improving integrated pest management (IPM) effectiveness.

Entomopathogenic fungi (EPF) have been shown to be effective biocontrol agents. Furthermore, they have been proven to cause a wide range of pre-mortality effects in infected insects, including mating behavior change, food consumption reduction, immune reaction alterations [5], and developmental rates, longevity, and fecundity reductions [6]. *Purpureocillium lilacinum* has primarily been described as a nematode pathogen, and later as a promising insect pest control agent over time. It has been reported to infect the Mexican fruit fly *Anastrepha ludens* (Lowe) (Diptera: Tephritidae) [7], the melon fly *Bactrocera cucurbitae* (Coquillett) (Diptera: Tephritidae) [8], the brown flour beetle *Tribolium confusum* Jacquelin du Val (Coleoptera: Tenebrionidae) [9], the cotton aphid *Aphis gossypii* Glover, 1877 (Hemiptera: Aphididae) [10], the greenhouse whitefly *Trialeurodes vaporariorum* (Westwood) (Aleyrodidae: Homoptera), the cotton bollworm *Helicoverpa zea* Boddie (Lepidoptera: Noctuidae) [11], the fruit fly *Bactrocera* spp. [12], the greater wax moth *Galleria mellonella* (Linnaeus 1758) (Lepidoptera: Pyralidae) [13], the South American tomato pinworm *Tuta absoluta* (Meyrick) (Lepidoptera: Gelechiidae) [14], the Asian citrus psyllid *Diaphorina citri* (Kuwayama) (Hemiptera: Psyllidae) [15], and the sweet potato whitefly *Bemisia tabaci* (Gennadius) (Hemiptera: Aleyrodidae) [16].

*Purpureocillium lilacinum* is a slow-killing pathogen that is able to survive in the host for a long period [17], which may increase its efficacy and its transmission rate [5, 7]. It is now well established that protease, phospholipase, chitinase, mannanase, beta-glucanase, and lipase hydrolyzing enzymes, play important roles in *P*. *lilacinum* pathogenicity [18]. This was also reported for leucinostatins and paecilomide secondary metabolites [19]. Many of these secondary metabolites were shown to modulate the host immune system, as well as insecticidal, cytotoxic, and antimicrobial properties [20].

A growing number of studies have proven that *P*. *lilacinum*, in addition to causing insect mortality, has negative effects on the host’s biological parameters [21], including reproduction [10, 20], growth [22], food consumption, and sexual behavior [23]. Meanwhile, the mechanisms of host-fungus interaction remain poorly understood. To enhance our knowledge of this pathogenic relationship, we investigated the pre-mortality activities of *P*. *lilacinum* on *C*. *capitata*, by analyzing (i) fertility and fecundity parameters, (ii) nutrient reserves, (iii) biochemical enzyme activities, and (iv) defense-related genes expression.

## Materials and methods

### Flies

The Mediterranean fruit flies used in the experiments were from a colony of the VIENNA 8 (V8) genetic sexing strain (GSS) maintained in the Tunisian Medfly rearing facility in the National Centre of Nuclear Sciences and Technology (CNSTN). This strain has two mutations with two markers: white pupae (*wp)* and temperature-sensitive lethal mutation (*tsl*). The mass rearing was carried out under optimal conditions [24, 25]. Wild flies were obtained after sampling infested fruits hosting the fly. The fruits were then collected and placed on a mesh screen in a plastic container, allowing larvae to emerge from the fruit and pupate at room temperature. The pupae collected in this way are placed in bottles for the emergence of adults, which will then be used in the bioassays.

### Fungal isolate

*Purpureocillium lilacinum* (Accession number: ON212642) was isolated from soil samples collected from a citrus orchard in Nabeul governorate, Tunisia (N 36°27′21″ E 10°44′15″, Al 14m). PCR amplicons were sequenced and similarity searches were realized by aligning the sequences obtained from GenBank with BLASTn (https://blast.ncbi.nlm.nih.gov/Blast.cgi).

### Bioassays

Medfly V8 was used in laboratory bioassays. The bioassays were monitored to determine *P*. *lilacinum*’s pathogenicity on the fruit fly. *Purpureocillium lilacinum* was evaluated on 5-day-old males and females, using abdominal topical treatments. After 6 days of incubation at 30°C, conidial production was examined. Conidia were dislodged by washing 10 mL with 0.02 percent Tween 80, and conidial concentration was determined using a hemocytometer. The final suspension concentrations were adjusted to 10^7^ conidia/mL, with sterile water. The bioassays were performed in eight replicates under optimal conditions of 20 ± 2°C, 50–60% RH, and a photoperiod of 15:9 (L:D) h. Fifteen individuals (males and females) were each given 5 μl of the specified suspensions, before being placed in a ventilated Petri dish (89 mm diameter, 23 mm height). Adult flies were fed on sugar, yeast (3:1), and water. Control flies were treated with Tween 80 (0.02%), which was free of fungal suspension.

### Fecundity and fertility parameters

Adult fecundity was measured to evaluate the effect of medfly adult exposure to *P*. *lilacinum* treatment through the bioassays method. Following the fungal suspension inoculation, flies were transferred to a ventilated Petri dish (89 mm diameter, 23 mm height) conidia-free, and 3 replicates were performed. Each replicate consists of a group of five females and five males: ♂ control X ♀ control; ♂ treated X ♀ treated. After 48 hours of treatment, we counted the total number of eggs laid on each replicate within 5 days [26]. Egg fertility was evaluated by lining up one hundred eggs in a Petri dish. Petri dishes are then incubated at 26°C with 60% RH and a photoperiod of 16:8 (L: D) h for 48h. The number of larvae hatched was counted for 7 days [26].

### Nutrient reserves measurement

To measure larval nutrient reserves, three third-instar larvae per replicate (3 replicates) were homogenized in 0.2 mL of Na_2_SO_4_ (2%) with the addition of 1.3 mL chloroform-methanol (1:2) (vol:vol) solution. After 10 min of centrifugation at 5200 g, the supernatant was used for lipid quantification and the pellet was further treated for glycogen and sugars estimation [27]. Lipid quantification was assayed as described previously by Warburg and Yuval [28]. After chloroform:methanol (1:2) total evaporation at 90°C, lipids were resuspended in 0.3 mL H_2_SO_4_. Lipids were hydrolyzed at 90°C for 10 min and an aliquot (30 μl) was reacted with the 270 μl vanillin reagent for 30 min at room temperature. Lipids were resuspended in 0.3 mL H_2_SO_4_ after entire evaporation in chloroform: methanol (1:2) at 90°C.

### Enzymatic assays

Assays were carried out after dissecting the digestive tracts of 50 adult flies per replicate (3 replicates) in NaCl (0.15 M). The intestines were homogenized in a 50 mM Tris-HCl buffer (pH 8.0). After centrifugation at 10000 g for 30 min at 4°C, the supernatant was collected and stored at -20°C, to be used for enzymatic activities [29]. Disaccharide and polysaccharide activity was determined according to Baker et al. [30]. Briefly, 80 μl of intestinal homogenates were incubated for 30 min at 37°C with 60 μl of 1% substrate solutions (maltose, sucrose, pectin, and starch) and 400 μl of 0.1 M sodium acetate buffer (pH = 5.5). After heating the aliquots in boiling water for 10 min, 100 μl of DNS solution were added. The absorbance was measured at 540 nm. One activity unit (AU) was defined as the amount of enzyme activity that increased absorbance by 0.01 at 540 nm.

The estimation of carbohydrase activity was determined using p-nitrophenol (PNP) released by hydrolysis of the corresponding p-nitrophenyl conjugates as substrates: PNP α-glu and PNP β-gal dissolved in sodium acetate buffer 0.1 M, pH = 5.5 [29]. The substrate solution 3 mM (3 μl) was incubated with 50 μl of the intestinal homogenates and 450 μl of sodium acetate buffer at 37°C for 10 min. The reaction was stopped by adding 130 μl of acetic acid (30%, p/v). The amount of nitrophenol released was estimated by measuring the absorbance at 405 nm. One activity unit (AU) was defined as the amount of enzyme activity that increased absorbance by 0.01 at 405 nm.

Protease activity was measured using azocasein as a substrate, according to Silva et al. [29]. Briefly, aliquots of 50 μl of intestinal homogenate were incubated with 450 μl of buffer solution (50 mM Tris-HCl, 20 mM CaCl_2_, pH 8.0) and 500 μl of azocasein solution (1.5%) at 37°C for 30 min. To stop the reaction, 150 μl of TCA solution (20%) were added. The samples were centrifuged at 10000 g for 10 min. The obtained supernatant was alkalinized with NaOH (0.2 N). The absorbance at 440 nm of soluble peptides was measured. One activity unit (AU) was defined as the amount of enzyme activity that increased absorbance by 0.01 at 440 nm. Hemoglobinase activity was measured as described in Silva et al. [29]. Intestinal homogenates (100 μl) were incubated with 50 μl sodium acetate buffer solution (0.2 M, pH 4.5) and hemoglobin solution (1%) at 37°C for 60 min before adding 100 μl of a TCA solution (40%). Samples were centrifuged at 10000 g for 10 min and the recovered supernatant was alkalinized with NaOH (2 N) solution. Soluble peptides were then measured at 750 nm using the Lowry technique [31]. The amount of enzyme activity that raised absorbance by 0.01 at 750 nm was defined as one activity unit (AU). All enzymatic experiments were carried out in triplicate. For the negative control, the gut homogenate was replaced with saline solution, and gut activities were expressed as activity units per gut (AU/gut).

### Molecular assays

RNA extraction was performed using Trizol reagent (Invitrogen) following the manufacturer’s procedures. Total RNAs were extracted using 3 adult flies per replicate (3 replicates) collected 0h, 24h, 72h, and 144h after inoculation of conidial suspension (10^7^ conidia/mL). RNA concentration and purity were assessed at 260 and 280 nm absorbance using a Nanodrop® spectrophotometer (Thermo Scientific). Total RNA was treated using the RQ1RNase -Free DNase kit (Promega). Purification of the RNA was subsequently performed using the Nucleospin RNA Clean-up kit (Macherey-Nagel). The synthesis of the cDNA was carried out with the Kit iScriptTM cDNA Synthesis (Bio-Rad) using 1 μg of RNA in 20 μl of reaction volume. The reaction program comprises a primer hybridization step (oligo (dT) and random) at 25°C for 5 min, a second RT step at 46°C for 20 min, and finally inactivation of the reaction at 95°C for 1 min.

Quantitative Real-Time PCR (qRT-PCR) analysis was performed to identify transcript changes in response to the infection of selected genes, using cDNA derived from total RNA of fly samples. Five genes encoding antimicrobial peptides (AMPs), namely *ceratotoxin-A*, *attacin-A*, *cecropin-1*, *takeout*, *defensin*, and two additional immune genes, namely *relish* and *pgrp-LC*, were analyzed, as well as two reference genes, namely *gapdh* and *g6pdh*, for the normalization (S1 Table) [32]. Conventional PCR reactions were first performed for the preparation of the standard ranges (making the standard curve). The PCR products were subsequently purified using the GenElute TM PCR Clean-Up Kit (Sigma-Aldrich). The real-time quantification was performed in a CFX Connect TM Real-Time System (Bio-Rad) using the MasterCycler® 480 SYBR Master I Master (ROCHE). Cycling conditions involved an initial 95°C for three minutes, 40 cycles of 10 seconds at 95°C, 30 seconds at 57°C and 30 seconds at 68°C. A fluorescence reading was made at the end of each extension step. Three replicates were performed and the specificity of the amplification products was assessed by melt-curve analysis. The standard curves were constructed with serial 6-fold dilutions for each AMP gene studied, ranging from 2000 fg/μl to 0.2 fg/μl. PCR efficiencies were above 94% for all primer pairs (S1 Table).

### Statistical analysis

Data were analyzed using R version 4.1.3 (2022-03-10) [33]. For the survival analysis, replicate data were pooled and survival curves were produced using the survminer R library. Treated versus untreated curves were compared using the Logrank test.

For the effect of *P*. *lilacinum* on *C*. *capitata* fecundity and fertility parameters, we used t-tests to compare the number of eggs laid by inoculated vs. control females and to compare the number of eggs hatched.

For the nutrient reserves measurement and enzymatic assays, data analysis was processed using single-factor ANOVA followed by Dunnett post-hoc tests controlling the global alpha risk to 5%. Results are reported as means ± standard error (SE).

Data from molecular assays were computed gene by gene, comparing relative gene expression between the infected and the uninfected groups across a complete factorial linear model including sex (male, female), mating status (virgin, mature, and mated), and t (24, 72 and 144 hours) as additional covariables. Relative expressions were log-transformed to assume the residual normality of the model. As some interactions were significant, post-hoc tests comparing infected versus uninfected groups were performed for each covariable combinations. The model, variance decomposition analysis, and post-hoc tests are presented in S1 Data.

## Results

### Bioassays

**Fig 1:**
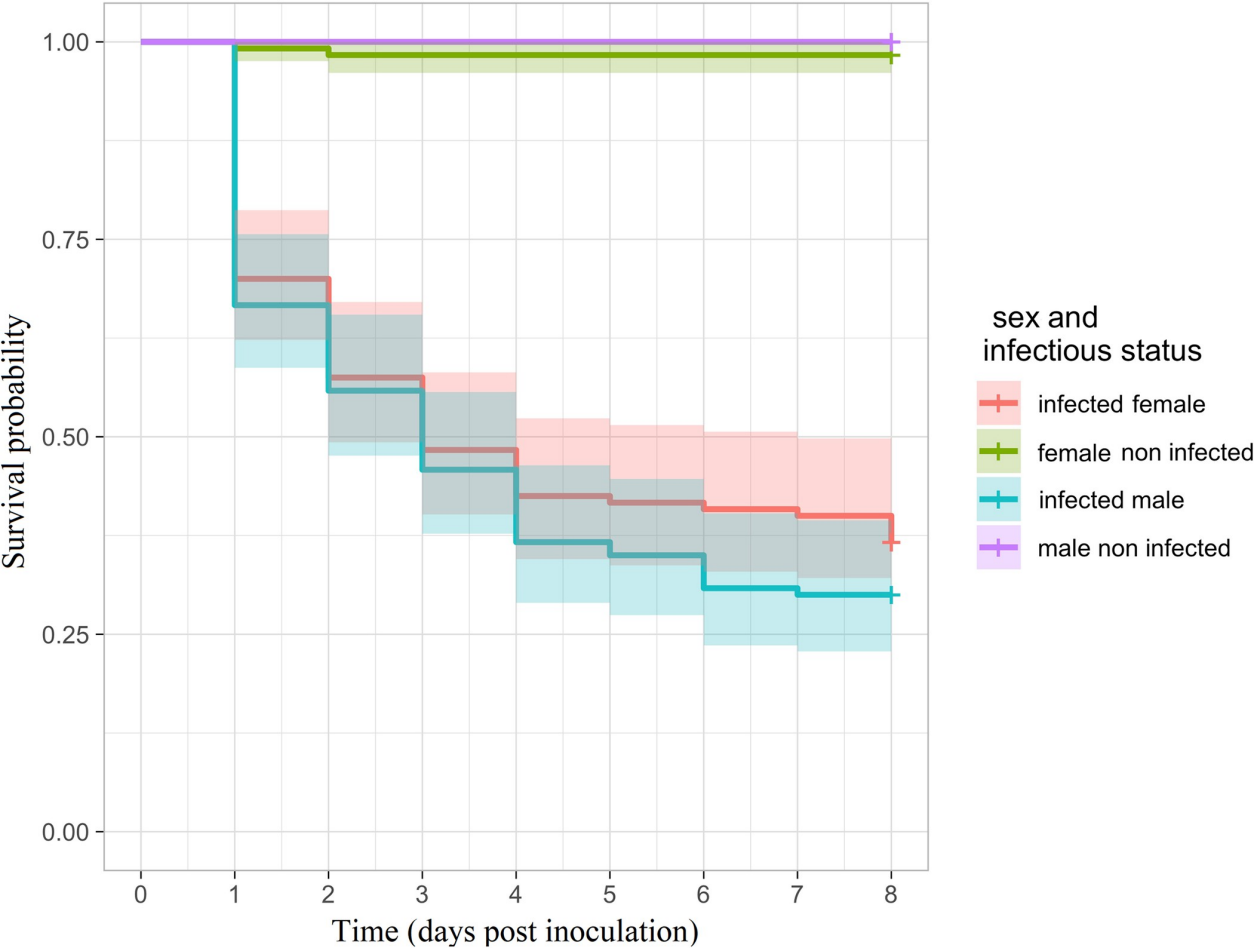
Survival analysis of *Ceratitis capitata* males and females, infected and non-infected (controls) with *P*. *lilacinum*.

*Ceratitis capitata* (V8) adults were individually treated by topical application of fungal suspension. Mortality was monitored for 8 days. *Purpureocillium lilacinum* caused 70.00±1.05% of mortality on males and 63.33±1.21% on females of *C*. *capitata* (V8) compared to the control (0.00% and 1.7±1.1%, respectively) after 8 days at 1.1x10^8^ conidia/mL (Fig 1). The survival difference between infected versus non-infected group is significant (log rank test, p = 2.10^−16^).

The 8 replicates of 15 flies for each condition were pooled. Survival curves are reported with their 95% confidence bands.

### Fecundity and fertility parameters

**Fig 2:**
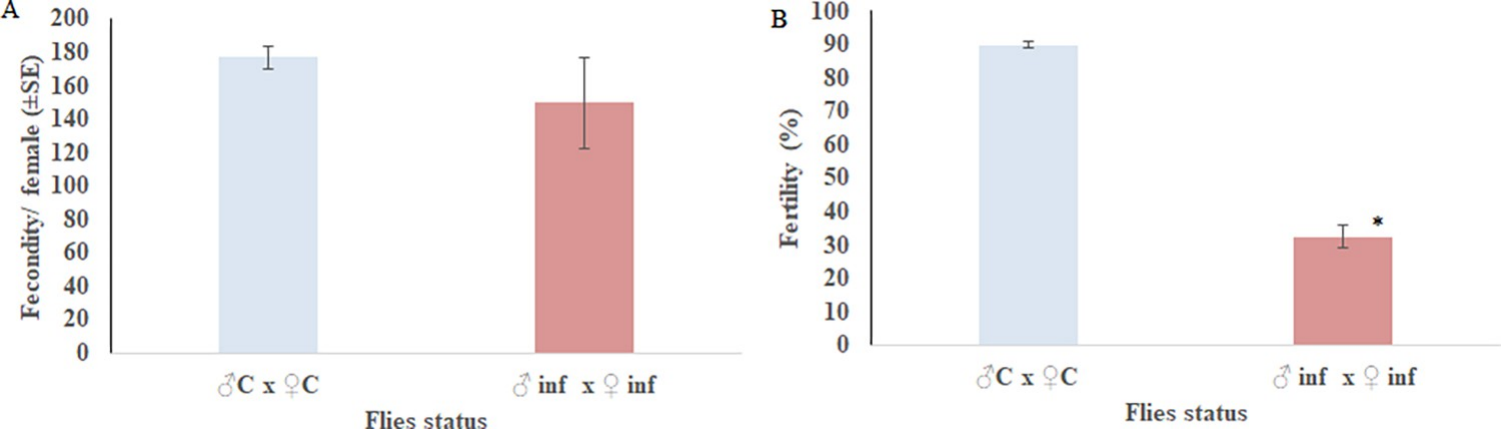
Effect of *P*. *lilacinum* on fecundity and fertility of *Ceratits capitata*. (A) Bar chart illustrating the mean number of eggs laid by non-infected and infected females of *C*. *capitata*; (B) Bar chart showing the mean proportion of eggs that hatched from eggs laid by non-infected and infected females of *C*. *capitata*. Error bars indicate the standard error. (*) indicates a significant difference between infected adults and non-infected (p<0.05).

The inoculation of the spore suspension did not significantly reduce the fecundity of *C*. *capitata* (Fig 2). However, a significant reduction in egg fertility (the number of larvae hatched) of treated females was observed. Egg fertility was reduced significantly by 57.59%: only 32.37±3.43% of the eggs hatched from infected flies compared to 89.96 ± 0.95% from non-infected flies (p = 0.002) (Fig 2).

### Nutrient reserves measurement

**Fig 3:**
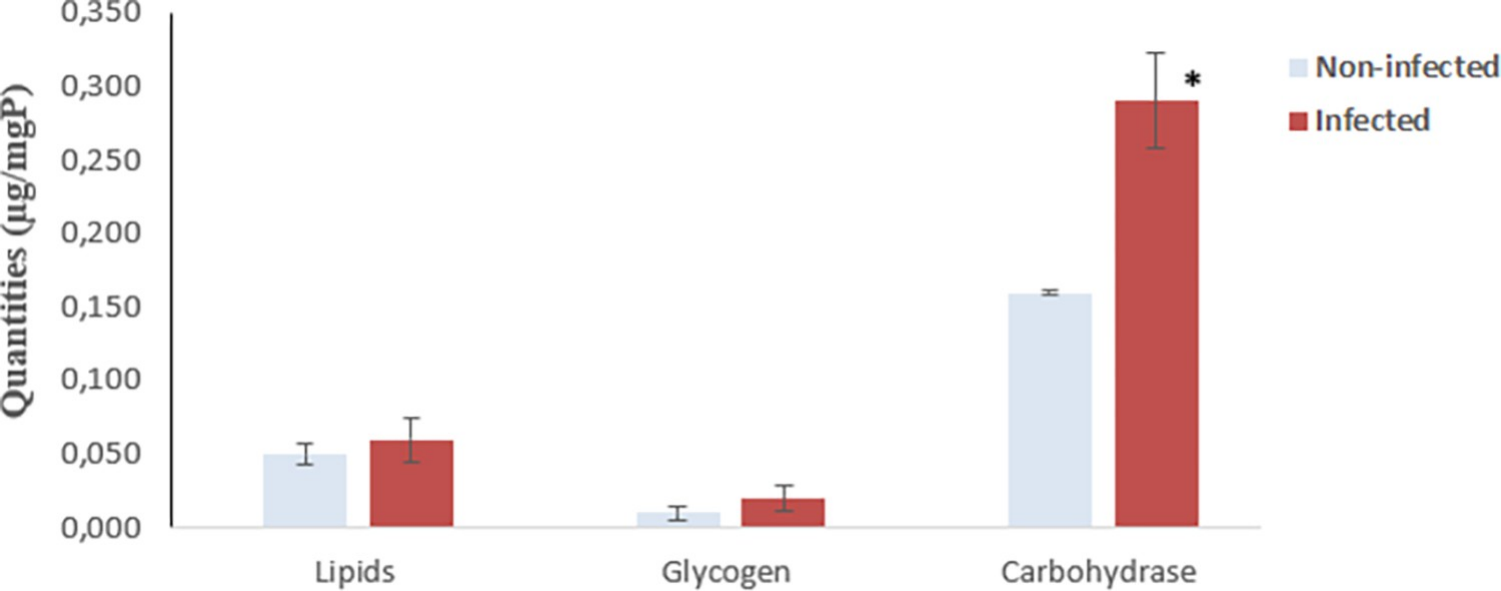
Average amounts of lipid, glycogen, and sugar in infected larvae (red) and non-infected larvae (blue) of *Ceratitis capitata*. Error bars indicate the standard error. (*) indicates a significant difference between columns (P<0.05).

Treatment with *P*. *lilacinum* resulted in a varying composition of larvae nutrient reserves. The amount of sugar increased significantly (p = 0.02) in tissues of larvae of third instars from infected flies with 0.521 ± 0.002 μg/ mg of protein, compared to larvae from non-infected flies with 0.332 ± 0.001 μg/ mgP. On the contrary, glycogen and lipid levels remained unchanged; there was no significant difference between larvae from infected and non-infected flies (Fig 3).

### Enzymatic assays

**Fig 4:**
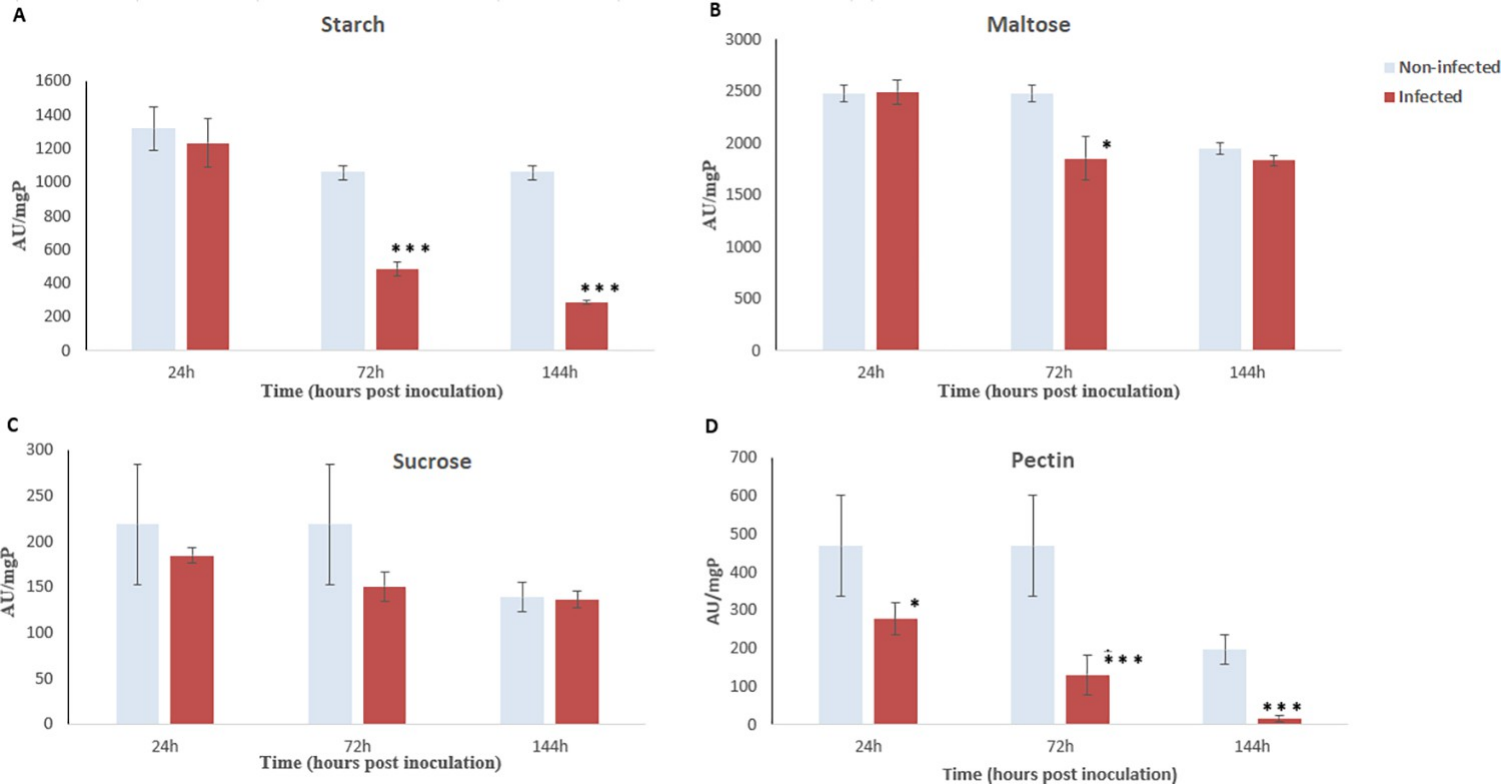
Digestive disaccharide and polysaccharide activities after fungal inoculation to *Ceratitis capitata* (24 to 144 hours post-inoculation). Blue and red bars chart illustrating enzymatic activities per mg protein for non-infected and infected flies respectively. (A) represent enzymatic activities on starch; (B) represent enzymatic activities on maltose (C) represent enzymatic activities on sucrose; (D) represent enzymatic activities on pectin. Error bars indicate the standard error. (* p<0.05, ** p<0.01, *** p<0.001).

The carbohydrase activity in whole midguts of infected flies showed a significant decrease post-inoculation for starch (487.04 ± 41.98 AU/mgP (p < 0,001)), for pectin (130.48 ± 51.95 AU/mgP (p = 0.001)), and for maltose (1856.93 ± 206.02 AU/mgP (p = 0.027)) compared to non-infected (1055.68 ± 44.29 AU/mgP, 468.37 ± 32.23 AU/mgP, and 2477.00 ± 82.73 AU/mgP, respectively) (Fig 4).

**Fig 5:**
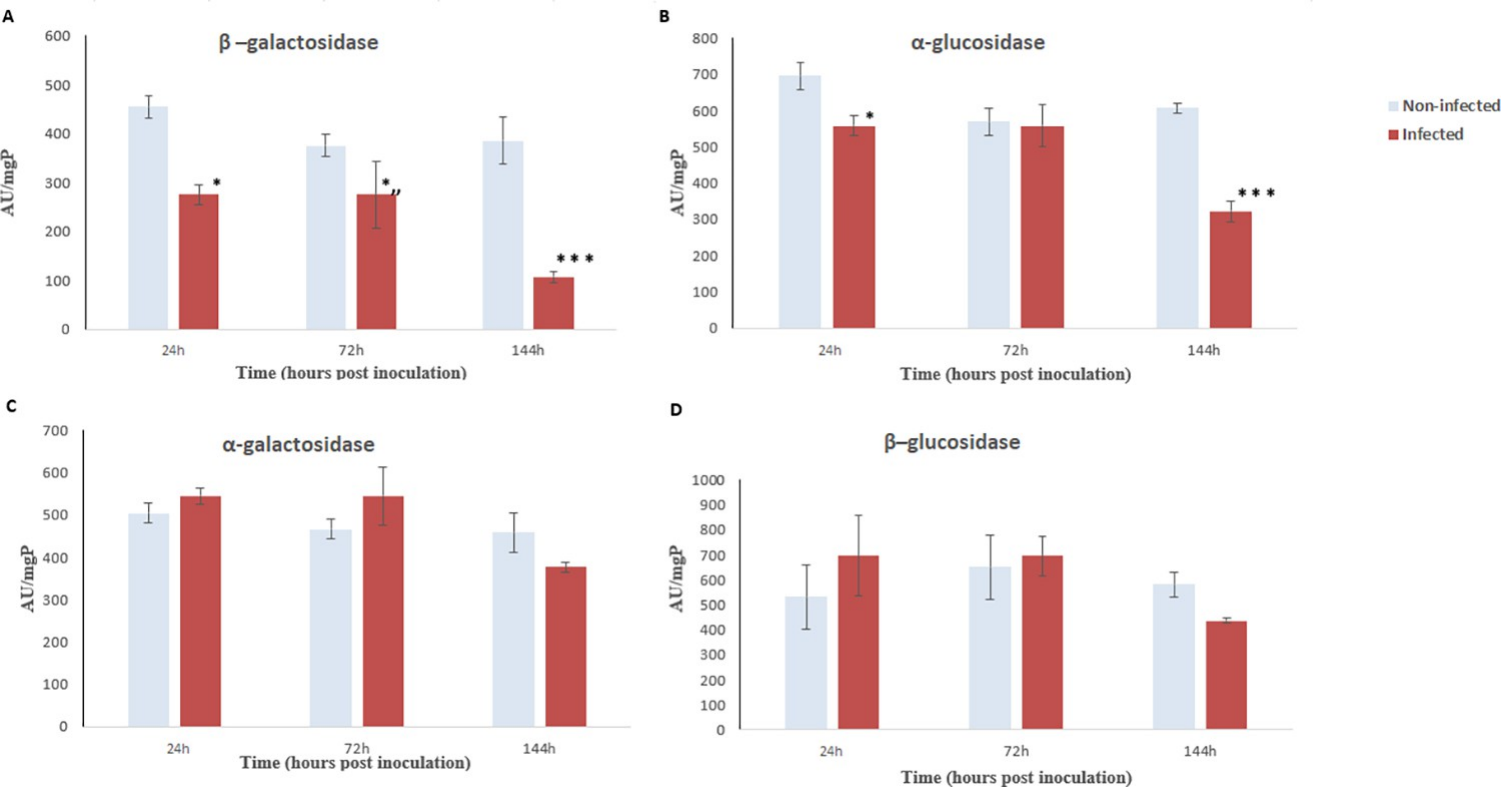
Digestive glycosyl hydrolase activities after fungal inoculation to *Ceratitis capitata* (24 to 144 hours post-inoculation). Blue and red bars chart represent glycosyl hydrolase activities mg protein for non-infected and infected flies respectively. (A) β –galactosidase activities; (B) α-glucosidase activities; (C) α-galactosidase activities and (D) β–glucosidase activities. Substrates used: PNPaglu, PNPbglu, PNPagal, PNPbgal. Error bars indicate the standard error. (* p<0.05, ** p<0.01, *** p<0.001).

The digestive glycosyl activity appears to be affected as well during infection. Infected flies showed a significant decrease in α-glucosidase specific activity (321.93 ± 14.92 AU/mgP), when compared to non-infected flies (607.93 ± 27.83 AU/mgP (p = 0.0001)) and in β-galactosidase activity (107.06 ± 17.45 AU/mgP), when compared to non-infected flies (386.52 ± 36.28 AU/mgP (p < 0.0001)) (Fig 5).

**Fig 6:**
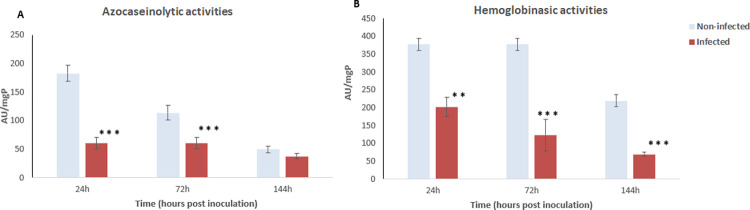
Digestive proteinase activities after fungal inoculation to *Ceratitis capitata* (24 to 144 hours post-inoculation). (A) Blue bar charts represent azocaseinolytic activities per mg of protein for non-infected flies; red bar charts represent azocaseinolytic activities per mg of protein for infected flies. (B) Blue bar charts represent hemoglobinasic activities per mg of protein for non-infected flies; red bar charts represent hemoglobinasic activities per mg of protein for infected flies. Error bars indicate the standard error. (* p<0.05, ** p<0.01, *** p<0.001).

The digestive proteinase (measured by the amounts of azocasein and hemoglobin hydrolysis in the midgut) was less active in infected adults of *C*. *capitata* than in non-infected flies. A higher capacity to digest protein in non-infected flies’ midgut was observed. Infection induced a significant decrease in azocaseinase (p = 0.001) and hemoglobinase (p = 0.024) activities by 37.48 ± 4.91 AU/mgP, and 69.24 ± 5.43 AU/mgP, respectively compared to non-infected (49.57 ± 5.58 AU/mgP, 219.41 ± 16.59 AU/mgP, respectively) (Fig 6).

### Molecular assays: Steady-state levels of gene transcripts

**Fig 7:**
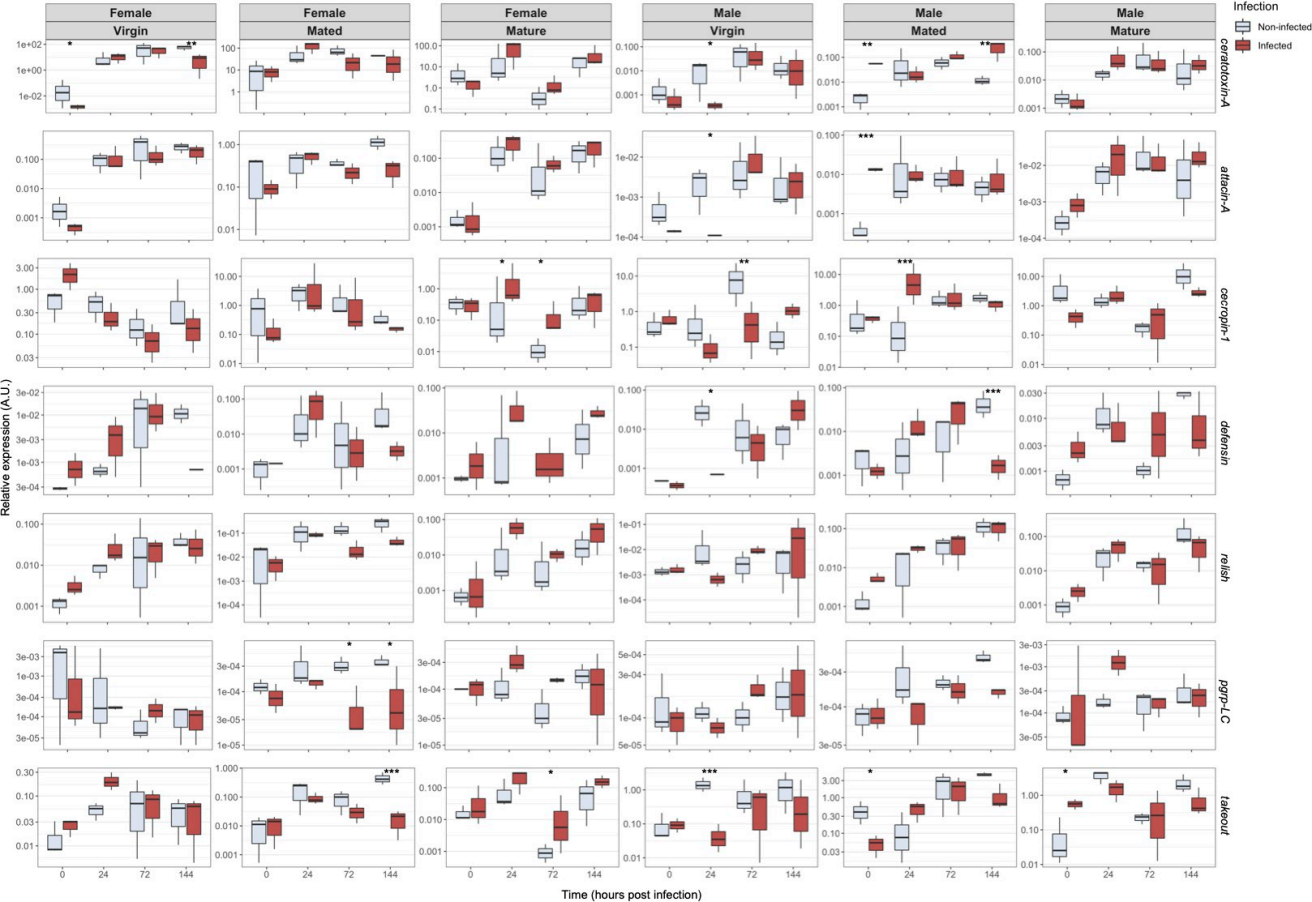
Expression of 7 immune genes in *Ceratitis capitata* inoculated with *P*. *lilacinum* for the times analyzed (0h, 24h, 72h, and 144h). The bottom and top “whiskers” represent minimum and maximum values, respectively. The thick line bisecting the box represents the median. The bottom and top of the box represent the 25th and 75th percentiles, respectively. Asterisks indicate a significant difference (* p<0.05, ** p<0.01, *** p<0.001) between treated and untreated control as given by the post hoc tests presented in S1 Data.

The expression profile of some AMPs encoding genes depends significantly on fly gender. Indeed, sex seems to strongly influence the expression of AMPs regardless of the infection status and other covariables. This is clearly highlighted by the scale changes observed in Fig 7 and the principal “sex” effects of the ANOVA (see S1 Data) for *ceratotoxin-A* (p = 2.10^−16^) *attacin-A* (p = 2.10^−16^), *cecropin-1* (p = 2.10^−5^) and *takeout* (p = 2.10^−16^).

The variable status (virgin, mature, and mated) also influenced the expression profile of some AMP encoding genes independently of infection and other covariables (Fig 7 and S1 Data). We noticed a significant activation of immune-response related genes for *ceratotoxin-A* (p = 10^−11^), *attacin-A* (p = 10^−9^), *cecropin-1* (p = 0.04), and the IMD pathway transcription factor *relish* (p = 10^−6^) for females (Fig 7). The expression profile of *attacin-A* and *ceratotoxin-A* increased with maturity compared to their counterparts virgin immature, and mated flies. Males showed a similar profile to females (Fig 7). Mature males expressed more AMPs than virgin immature and mated ones.

Time after infection induced a significant increase on 5 out of 7 transcripts studied (Fig 7 and S1 Data): *ceratotoxin-A* (p = 2.10^−16^), *attacin-A* (p = 2.10^−16^), *defensin* (p = 10^−7^), *relish* (p = 8.10^−14^) and *takeout* (p = 2.10^−9^).

Nonetheless, the infection factor was not significant as a principal effect in the factorial analysis (S1 Data) meaning that there was not an overall effect of infection that would increase or decrease the expression of the 7 studied genes of our study, independently of the included covariables. However, several differences in expressions were observed when comparing infected versus uninfected flies in some combinations of the covariables (Fig 7). Hence, a significant decrease on *ceratotoxin-A* 144 h post- inoculation (hpi) was observed for virgin females (p = 0.009), also within *pgrp-LC* (p = 0.022) *and takeout* (p = 0.0007) for mated females. On the contrary, an increase in transcript levels during maturation implies *cecropin-1* at 24 hpi (p = 0.039) and at 72 hpi (p = 0.026) and *takeout* (p = 0.45) at 72 hpi. For virgin males, we revealed that transcripts levels decreased at 24 hpi on *ceratotoxin-A* (p = 0.02), *attacin-A* (p = 0.01), *defensin* (p = 0.041), *takeout* (p = 0.0005), and at 72 hpi on *cecropin-1* (p = 0.0059). However, mated and infected males revealed increase in expression levels at time of infection (0 hpi) of *ceratotoxin-A* (p = 0.02) and *attacin-A* (p = 0.0003), and at 24 hpi of *cecropin-1* (p = 0.0004).

## Discussion

The purpose of this study was to analyze medfly biological processes that are affected by *P*. *lilacinum* infection, as well as to provide preliminary data for understanding the key mechanisms used by this entomopathogen. The pathogen infection may induce defense mechanisms, which in turn may affect the host’s behavioral and physiological responses. Therefore, we have screened the reproductive-based response (fecundity and fertility parameters), metabolic-based response (enzymatic activities), and molecular-based response (transcriptional profiles) of *C*. *capitata*.

*Purpureocillium lilacinum* showed an entomopathogenic effect against *C*. *capitata* adults. Likewise, similar results have been recently obtained with the Mexican fruit fly *A*. *ludens* [7] and the melon fly *B*. *cucurbitae* [8].

*Purpureocillium lilacinum* exhibits a broad range of pre-mortality effects. *Purpureocillium lilacinum* isolate reduces egg fertility. Our results are in agreement with those found by Toledo-Hernández et al. [7] who investigated the virulence and pre-mortality effects of *P*. *lilacinum* against the Mexican fruit fly *A*. *ludens*. Similar effects on *C*. *capitata* were obtained after infection with the fungi *Beauveria bassiana*, *Metarhizium anisopliae*, and *Paecilomyces fumosoroseus* [26, 34]. Moreover, we showed an increase in sugar content during the third larval instar of all sampled larvae from infected flies, when compared to similar low lipids and glycogen levels. Insect metamorphosis requires the consumption of carbohydrate reserves for the synthesis of pupal cuticle and imaginal tissues [35]. This suggests that the high sugar content measured following pathogen infection may be due to a lack of energy expenditure or to a defect in sugar digestion machinery (inhibition of the assimilation of sugars). Additional investigations are needed to test these hypotheses.

Enzymatic activities from *C*. *capitata* adults were also significantly impacted by infection under the *in vitro* assays (*i*.*e*., at optimum pH). *Ceratitis capitata* relies on specific α- and β-galactosidases associated with enzymatic activities on maltose and sucrose to digest carbohydrates, as well as complex proteolytic systems for protein digestion, especially the serine endoproteinase [29]. We showed that infection disrupts the capacity to digest carbohydrates, as measured by the amounts of di and polysaccharides in midguts. By the third day following infection, infected flies always exhibit a significant decrease of their enzymatic activities, when compared to non-infected flies. These findings are consistent with those reported by Malaikozhundan and Vinodhini [36] on the infection of the cowpea weevil *Callosobruchus maculatus* (Fabricius 1775) (Coleoptera: Bruchidae) with *B*. *thuringiensis*, and, suggest that the metabolism activities decrease within *C*. *capitata* is a direct effect of *P*. *lilacinum* enzymes.

Sugars and proteins are macronutrients that play critical physiological roles in Diptera resource allocation, longevity [37], reproduction [27, 38], larval development [39], mating success [40], and sex pheromone production [41]. Digestion and absorption of carbohydrates stimulate lipogenic activity (incorporation of lipid reserves). Lipids modulate hormonal changes such as juvenile hormone (JH) [42], which regulates mature sexual behavior in both males and females of diverse insect orders [43]. Furthermore, it has been proven that limiting access to host fruits and dietary protein delays or suppresses the egg-laying ability of laboratory-reared females of both *C*. *capitata* (Wiedmann) and *B*. *cucurbitae* (Coquillett) [44]. Adults of *C*. *capitata* can allocate energetic reserves to be used slowly under a ‘‘reproductive waiting mode” [45] or/and progress to egg-reabsorption in reproductive females as observed in the olive fly *Bactrocera oleae* (Rossi) (Diptera: Tephritidae) [27]. Based on these findings, we may conclude that *P*. *lilacinum* infection causes reduced carbohydrate digestion (after enzymatic inhibition), which is associated with decreased fertility and fecundity in *C*. *capitata*.

The immune system is a key barrier against viruses, bacteria, fungi, and parasite infections, and therefore nutrition is essential for insect resistance to these infections [46–48]. According to Gomulski et al. [32], the abundance of immune gene transcripts within *C*. *capitata* may be related to the nature of the food sources. In our case, the apparent lack of immune response following fungal inoculation in *C*. *capitata* may be related to the enzymatic activities decrease in inoculated flies.

Aging causes physiological changes in the immune system response. Previous studies have shown that AMPs amount increased with age in *Drosophila* [49–53] and honey bees *Apis mellifera* (Linnaeus) (Himenoptera: Apidae) [54]. The up-regulation observed with *attacin-A*, *takeout*, *pgrp-LC*, *cecropin-1*, *ceratotoxin-A*, and *defensin* genes is most likely due to involvement in other physiological mechanisms occurring concurrently during infection, rather than the proper effect of infection, as it was also observed by Gomulski et al. [32]. Within *C*. *capitata*, a process of immunity anticipation prior to mating has already been reported [32]. When compared to their immature virgin counterparts, *C*. *capitata* produced significantly more immune-response related genes (*attacin-A*, *ceratotoxin-A*, *relish*, and *cecropin-1*).

Regardless of the infection challenge, the effect of sex on the expression of specific *C*. *capitata* AMP genes appears to be significant. Females and males express different transcriptomic profiles of *attacin-A*, *ceratotoxin-A*, *takeout*, and *cecropin-1*. This sex-specific response was shown previously with *C*. *capitata* and *Drosophila suzukii* (Matsumura) (Diptera: Drosophilidae) challenged with spinosad [55, 56].

Additionally, fungi are known to produce secondary metabolites that display various immunosuppressive properties capable to disrupt the host’s physiological processes [57]. Several fungi exhibited a potential to modulate the immune response of different insects, including the large pine weevil, *Hylobius abietis* (Linnaeus, 1758) (Coleoptera: Curculionidae) [58], the greater wax moth, *Galleria mellonella* (Lepidoptera: Pyralidae) (Linnaeus, 1758) [59], and the tobacco caterpillar, *Spodoptera litura* (Fabricius) (Lepidoptera: Noctuidae) [60]. *Purpureocillium lilacinum* is known to produce leucinostatins, a family of lipopeptide antibiotics with broad biological activity, including nematode mortality and reproduction inhibition [20]. These findings drew our attention to the relationships between the down regulation observed on *ceratotoxin-A*, *attacin-A*, *defensin*, *pgrp-lc*, *takeout*, and *cecropin-1* genes and leucinostatins. We hypothesize that *P*. *lilacinum* uses leucinostatins as immune system inhibitors to maximize its proliferation rate.

The effects stated above, in our opinion, may lie beneath the remarkable effects caused by *P*. *lilacinum*. A cause-and-effect relationship between fungal inoculation and metabolic and molecular disorders was discovered. These findings provide comprehensive insight into the dynamics of the medfly’s response to *P*. *lilacinum* infection, revealing that metabolic processes, rather than immunological functions, played a key part in the response. Furthermore, metabolic and molecular information about *C*. *capitata*’s response systems to *P*. *lilacinum* proliferation, of which little is known to date, could lead to a better understanding of the mechanism in controlling plant pests, as well as to improve its capabilities as a biocontrol agent.

## Conclusion

The current study provides a preliminary data for understanding the machinery displayed behind entomopathogen inoculation to *C*. *capitata*. This work helped to identify key physiological mechanisms targeted by *P*. *lilacinum* pathogen, opening a new perspectives for future medfly pest management. Our findings also provide evidence that the entomopathogenic fungus not only affect *C*. *capitata* immunity, but also mediate the metabolic machinery.

## Supporting information

S1 Table Primers sequences used in q-PCR. (DOCX)

S1 Data Variance Analysis tables of the complete linear models for the 7 genes of the study. (DOCX)
