# Supplementary material for: Differential responses of Ceratitis capitata to infection by the entomopathogenic fungus Purpureocillium lilacinum
Source: PLoS One. 2023 Sep 28;18(9):e0286108. doi: 10.1371/journal.pone.0286108 (PMC10538767; doi:10.1371/journal.pone.0286108)
Supplement: S1 Table — (DOCX) [file pone.0286108.s001.docx]

| Sequence ID | Forward primer | Reverse primer | References | |
| --- | --- | --- | --- | --- |
| X70020 (*Cecropin 1*) | gcgggttggctgaagaag | cggtggctgcgacattag | | [31] |
| AJ272446 (Ceratotoxin A) | gtggttaaacggagtattggtagc | aacgggtagagcagcctttg | | [31] |
| FC614 (*Attacin A*) | aaagtgtctacctctcgtttctgg | gcatagtagccactcaagtatcgc | [31] | |
| FS831 (*GAPDH2*) | ggtcgcatcggtcgtctgg | gctgaaacggtgcccttgaaac | [31] | |
| S67872 (*G6PDH*) | cggacgagcaggcaaaatatg | agacggacggcggtaagg | [31] | |
| HC1181 (*Defensin*) | ttggaatcctctgtgctttatgc | cgctgtgatttacgccgaag | [31] | |
| FC1457 (*Relish*) | acaaagttctcaatgcccacaatg | gttccttaacagcgatatgtagtgc | [31] | |
| HC731 (*PGRP-LC*) | gcacacaccaaaggctacaatc | cacccaaacgaagaccctcatc | [31] | |
| HC321 (*takeout*) | taaagcaagaggattcggcaaag | cccacccattgaagtatcatatcg | [31] | |
| HC1181 (*Defensin*) | ttggaatcctctgtgctttatgc | cgctgtgatttacgccgaag | [31] | |
