# Supplementary material for: Differential responses of Ceratitis capitata to infection by the entomopathogenic fungus Purpureocillium lilacinum
Source: PLoS One. 2023 Sep 28;18(9):e0286108. doi: 10.1371/journal.pone.0286108 (PMC10538767; doi:10.1371/journal.pone.0286108)
Supplement: S1 Data — (DOCX) [file pone.0286108.s002.docx]

**A - log(Ceratotoxin.A) ~ Status * Sex * Time * Infection**

**A.1 - Analysis of Variance Table**

Response: log(Ceratotoxin.A)

Df Sum Sq Mean Sq F value Pr(>F)

Status 2 109.60 54.80 31.0489 4.440e-11 ***

Sex 1 1313.18 1313.18 744.0364 < 2.2e-16 ***

Time 3 240.97 80.32 45.5113 < 2.2e-16 ***

Infection 1 0.23 0.23 0.1320 0.717194

Status:Sex 2 5.06 2.53 1.4332 0.243705

Status:Time 6 106.91 17.82 10.0953 1.357e-08 ***

Sex:Time 3 44.39 14.80 8.3830 5.382e-05 ***

Status:Infection 2 20.07 10.03 5.6854 0.004668 **

Sex:Infection 1 2.94 2.94 1.6663 0.199926

Time:Infection 3 0.70 0.23 0.1321 0.940758

Status:Sex:Time 6 78.10 13.02 7.3756 1.756e-06 ***

Status:Sex:Infection 2 6.56 3.28 1.8577 0.161721

Status:Time:Infection 6 22.72 3.79 2.1459 0.055227 .

Sex:Time:Infection 3 18.23 6.08 3.4425 0.019886 *

Status:Sex:Time:Infection 6 8.90 1.48 0.8408 0.541544

Residuals 94 165.90 1.76

---

Signif. codes: 0 ‘***’ 0.001 ‘**’ 0.01 ‘*’ 0.05 ‘.’ 0.1 ‘ ’ 1

**A.2 - emmeans results**

Status = Virgin, Sex = Female, Time = 0:

contrast ratio SE df null t.ratio p.value

(Non-infected) / Infected 10.8512 11.7705 94 1 2.198 0.0304

Status = Mated, Sex = Female, Time = 0:

contrast ratio SE df null t.ratio p.value

(Non-infected) / Infected 0.4683 0.5079 94 1 -0.699 0.4860

Status = Mature, Sex = Female, Time = 0:

contrast ratio SE df null t.ratio p.value

(Non-infected) / Infected 3.2720 3.5492 94 1 1.093 0.2773

Status = Virgin, Sex = Male, Time = 0:

contrast ratio SE df null t.ratio p.value

(Non-infected) / Infected 2.2954 2.4899 94 1 0.766 0.4456

Status = Mated, Sex = Male, Time = 0:

contrast ratio SE df null t.ratio p.value

(Non-infected) / Infected 0.0350 0.0379 94 1 -3.092 0.0026

Status = Mature, Sex = Male, Time = 0:

contrast ratio SE df null t.ratio p.value

(Non-infected) / Infected 1.4259 1.7293 94 1 0.293 0.7705

Status = Virgin, Sex = Female, Time = 24:

contrast ratio SE df null t.ratio p.value

(Non-infected) / Infected 0.6130 0.6650 94 1 -0.451 0.6529

Status = Mated, Sex = Female, Time = 24:

contrast ratio SE df null t.ratio p.value

(Non-infected) / Infected 0.3795 0.4116 94 1 -0.893 0.3740

Status = Mature, Sex = Female, Time = 24:

contrast ratio SE df null t.ratio p.value

(Non-infected) / Infected 0.2265 0.2457 94 1 -1.369 0.1743

Status = Virgin, Sex = Male, Time = 24:

contrast ratio SE df null t.ratio p.value

(Non-infected) / Infected 15.8164 19.1815 94 1 2.277 0.0251

Status = Mated, Sex = Male, Time = 24:

contrast ratio SE df null t.ratio p.value

(Non-infected) / Infected 1.7304 1.8770 94 1 0.506 0.6144

Status = Mature, Sex = Male, Time = 24:

contrast ratio SE df null t.ratio p.value

(Non-infected) / Infected 0.2999 0.3253 94 1 -1.110 0.2697

Status = Virgin, Sex = Female, Time = 72:

contrast ratio SE df null t.ratio p.value

(Non-infected) / Infected 0.9717 1.0540 94 1 -0.026 0.9789

Status = Mated, Sex = Female, Time = 72:

contrast ratio SE df null t.ratio p.value

(Non-infected) / Infected 4.2335 4.5922 94 1 1.330 0.1866

Status = Mature, Sex = Female, Time = 72:

contrast ratio SE df null t.ratio p.value

(Non-infected) / Infected 0.2662 0.2888 94 1 -1.220 0.2255

Status = Virgin, Sex = Male, Time = 72:

contrast ratio SE df null t.ratio p.value

(Non-infected) / Infected 0.8567 0.9293 94 1 -0.143 0.8869

Status = Mated, Sex = Male, Time = 72:

contrast ratio SE df null t.ratio p.value

(Non-infected) / Infected 0.5187 0.5626 94 1 -0.605 0.5465

Status = Mature, Sex = Male, Time = 72:

contrast ratio SE df null t.ratio p.value

(Non-infected) / Infected 1.3757 1.4922 94 1 0.294 0.7694

Status = Virgin, Sex = Female, Time = 144:

contrast ratio SE df null t.ratio p.value

(Non-infected) / Infected 17.5612 19.0490 94 1 2.642 0.0097

Status = Mated, Sex = Female, Time = 144:

contrast ratio SE df null t.ratio p.value

(Non-infected) / Infected 2.5675 2.7850 94 1 0.869 0.3869

Status = Mature, Sex = Female, Time = 144:

contrast ratio SE df null t.ratio p.value

(Non-infected) / Infected 0.4440 0.4816 94 1 -0.748 0.4560

Status = Virgin, Sex = Male, Time = 144:

contrast ratio SE df null t.ratio p.value

(Non-infected) / Infected 1.5612 1.6934 94 1 0.411 0.6823

Status = Mated, Sex = Male, Time = 144:

contrast ratio SE df null t.ratio p.value

(Non-infected) / Infected 0.0546 0.0592 94 1 -2.681 0.0087

Status = Mature, Sex = Male, Time = 144:

contrast ratio SE df null t.ratio p.value

(Non-infected) / Infected 0.5244 0.5688 94 1 -0.595 0.5532

Tests are performed on the log scale

**B - log(Attacin.A) ~ Status * Sex * Time * Infection**

**B.1 - Analysis of Variance Table**

Response: log(Attacin.A)

Df Sum Sq Mean Sq F value Pr(>F)

Status 2 64.14 32.07 23.7457 4.843e-09 ***

Sex 1 329.50 329.50 243.9907 < 2.2e-16 ***

Time 3 207.72 69.24 51.2698 < 2.2e-16 ***

Infection 1 0.62 0.62 0.4556 0.5013942

Status:Sex 2 22.90 11.45 8.4802 0.0004167 ***

Status:Time 6 44.07 7.35 5.4391 7.502e-05 ***

Sex:Time 3 25.02 8.34 6.1767 0.0007162 ***

Status:Infection 2 8.67 4.33 3.2096 0.0449334 *

Sex:Infection 1 2.94 2.94 2.1780 0.1434086

Time:Infection 3 0.46 0.15 0.1145 0.9513974

Status:Sex:Time 6 13.38 2.23 1.6517 0.1419145

Status:Sex:Infection 2 4.52 2.26 1.6726 0.1934232

Status:Time:Infection 6 13.00 2.17 1.6047 0.1546636

Sex:Time:Infection 3 9.29 3.10 2.2919 0.0833176 .

Status:Sex:Time:Infection 6 7.00 1.17 0.8634 0.5249842

Residuals 92 124.24 1.35

---

Signif. codes: 0 ‘***’ 0.001 ‘**’ 0.01 ‘*’ 0.05 ‘.’ 0.1 ‘ ’ 1

**B.2 – emmeans results**

Status = Virgin, Sex = Female, Time = 0:

contrast ratio SE df null t.ratio p.value

(Non-infected) / Infected 3.7530 3.5611 92 1 1.394 0.1667

Status = Mated, Sex = Female, Time = 0:

contrast ratio SE df null t.ratio p.value

(Non-infected) / Infected 1.2226 1.1600 92 1 0.212 0.8327

Status = Mature, Sex = Female, Time = 0:

contrast ratio SE df null t.ratio p.value

(Non-infected) / Infected 1.1277 1.0700 92 1 0.127 0.8995

Status = Virgin, Sex = Male, Time = 0:

contrast ratio SE df null t.ratio p.value

(Non-infected) / Infected 3.1247 3.3149 92 1 1.074 0.2856

Status = Mated, Sex = Male, Time = 0:

contrast ratio SE df null t.ratio p.value

(Non-infected) / Infected 0.0276 0.0262 92 1 -3.785 0.0003

Status = Mature, Sex = Male, Time = 0:

contrast ratio SE df null t.ratio p.value

(Non-infected) / Infected 0.3317 0.3854 92 1 -0.950 0.3448

Status = Virgin, Sex = Female, Time = 24:

contrast ratio SE df null t.ratio p.value

(Non-infected) / Infected 0.8609 0.8168 92 1 -0.158 0.8749

Status = Mated, Sex = Female, Time = 24:

contrast ratio SE df null t.ratio p.value

(Non-infected) / Infected 0.6110 0.5797 92 1 -0.519 0.6049

Status = Mature, Sex = Female, Time = 24:

contrast ratio SE df null t.ratio p.value

(Non-infected) / Infected 0.5056 0.4797 92 1 -0.719 0.4741

Status = Virgin, Sex = Male, Time = 24:

contrast ratio SE df null t.ratio p.value

(Non-infected) / Infected 15.8881 16.8548 92 1 2.607 0.0107

Status = Mated, Sex = Male, Time = 24:

contrast ratio SE df null t.ratio p.value

(Non-infected) / Infected 0.9055 0.8592 92 1 -0.105 0.9169

Status = Mature, Sex = Male, Time = 24:

contrast ratio SE df null t.ratio p.value

(Non-infected) / Infected 0.4038 0.3832 92 1 -0.956 0.3418

Status = Virgin, Sex = Female, Time = 72:

contrast ratio SE df null t.ratio p.value

(Non-infected) / Infected 1.4228 1.3500 92 1 0.372 0.7110

Status = Mated, Sex = Female, Time = 72:

contrast ratio SE df null t.ratio p.value

(Non-infected) / Infected 1.7397 1.6507 92 1 0.584 0.5610

Status = Mature, Sex = Female, Time = 72:

contrast ratio SE df null t.ratio p.value

(Non-infected) / Infected 0.4112 0.3902 92 1 -0.937 0.3515

Status = Virgin, Sex = Male, Time = 72:

contrast ratio SE df null t.ratio p.value

(Non-infected) / Infected 0.4655 0.4417 92 1 -0.806 0.4225

Status = Mated, Sex = Male, Time = 72:

contrast ratio SE df null t.ratio p.value

(Non-infected) / Infected 0.8001 0.7592 92 1 -0.235 0.8147

Status = Mature, Sex = Male, Time = 72:

contrast ratio SE df null t.ratio p.value

(Non-infected) / Infected 1.2295 1.1666 92 1 0.218 0.8281

Status = Virgin, Sex = Female, Time = 144:

contrast ratio SE df null t.ratio p.value

(Non-infected) / Infected 1.5617 1.4818 92 1 0.470 0.6396

Status = Mated, Sex = Female, Time = 144:

contrast ratio SE df null t.ratio p.value

(Non-infected) / Infected 4.8056 4.5598 92 1 1.654 0.1015

Status = Mature, Sex = Female, Time = 144:

contrast ratio SE df null t.ratio p.value

(Non-infected) / Infected 0.7390 0.7012 92 1 -0.319 0.7506

Status = Virgin, Sex = Male, Time = 144:

contrast ratio SE df null t.ratio p.value

(Non-infected) / Infected 0.9852 0.9348 92 1 -0.016 0.9875

Status = Mated, Sex = Male, Time = 144:

contrast ratio SE df null t.ratio p.value

(Non-infected) / Infected 0.6282 0.5961 92 1 -0.490 0.6253

Status = Mature, Sex = Male, Time = 144:

contrast ratio SE df null t.ratio p.value

(Non-infected) / Infected 0.2550 0.2419 92 1 -1.440 0.1532

Tests are performed on the log scale

**C - log(Cecropin.1) ~ Status * Sex * Time * Infection**

**C.1 - Analysis of Variance Table**

Response: log(Cecropin.1)

Df Sum Sq Mean Sq F value Pr(>F)

Status 2 10.085 5.0425 3.1162 0.048839 *

Sex 1 30.497 30.4972 18.8470 3.509e-05 ***

Time 3 9.816 3.2719 2.0220 0.115949

Infection 1 0.002 0.0022 0.0014 0.970407

Status:Sex 2 15.295 7.6476 4.7261 0.011024 *

Status:Time 6 74.264 12.3773 7.6491 9.987e-07 ***

Sex:Time 3 23.385 7.7951 4.8173 0.003618 **

Status:Infection 2 2.476 1.2378 0.7649 0.468184

Sex:Infection 1 0.300 0.3004 0.1856 0.667551

Time:Infection 3 6.900 2.3001 1.4215 0.241272

Status:Sex:Time 6 18.005 3.0008 1.8544 0.096633 .

Status:Sex:Infection 2 15.604 7.8022 4.8217 0.010106 *

Status:Time:Infection 6 35.681 5.9468 3.6751 0.002496 **

Sex:Time:Infection 3 5.151 1.7169 1.0610 0.369426

Status:Sex:Time:Infection 6 11.444 1.9073 1.1787 0.324163

Residuals 96 155.342 1.6181

---

Signif. codes: 0 ‘***’ 0.001 ‘**’ 0.01 ‘*’ 0.05 ‘.’ 0.1 ‘ ’ 1

**C.2 – emmeans results**

Status = Virgin, Sex = Female, Time = 0:

contrast ratio SE df null t.ratio p.value

(Non-infected) / Infected 0.2393 0.2486 96 1 -1.377 0.1718

Status = Mated, Sex = Female, Time = 0:

contrast ratio SE df null t.ratio p.value

(Non-infected) / Infected 2.7599 2.8666 96 1 0.977 0.3308

Status = Mature, Sex = Female, Time = 0:

contrast ratio SE df null t.ratio p.value

(Non-infected) / Infected 1.2132 1.2600 96 1 0.186 0.8528

Status = Virgin, Sex = Male, Time = 0:

contrast ratio SE df null t.ratio p.value

(Non-infected) / Infected 0.6063 0.6298 96 1 -0.482 0.6311

Status = Mated, Sex = Male, Time = 0:

contrast ratio SE df null t.ratio p.value

(Non-infected) / Infected 0.8685 0.9020 96 1 -0.136 0.8923

Status = Mature, Sex = Male, Time = 0:

contrast ratio SE df null t.ratio p.value

(Non-infected) / Infected 7.8254 8.1277 96 1 1.981 0.0505

Status = Virgin, Sex = Female, Time = 24:

contrast ratio SE df null t.ratio p.value

(Non-infected) / Infected 1.9395 2.0145 96 1 0.638 0.5251

Status = Mated, Sex = Female, Time = 24:

contrast ratio SE df null t.ratio p.value

(Non-infected) / Infected 0.8725 0.9062 96 1 -0.131 0.8958

Status = Mature, Sex = Female, Time = 24:

contrast ratio SE df null t.ratio p.value

(Non-infected) / Infected 0.1146 0.1190 96 1 -2.086 0.0397

Status = Virgin, Sex = Male, Time = 24:

contrast ratio SE df null t.ratio p.value

(Non-infected) / Infected 4.0844 4.2422 96 1 1.355 0.1786

Status = Mated, Sex = Male, Time = 24:

contrast ratio SE df null t.ratio p.value

(Non-infected) / Infected 0.0217 0.0225 96 1 -3.689 0.0004

Status = Mature, Sex = Male, Time = 24:

contrast ratio SE df null t.ratio p.value

(Non-infected) / Infected 0.6567 0.6821 96 1 -0.405 0.6865

Status = Virgin, Sex = Female, Time = 72:

contrast ratio SE df null t.ratio p.value

(Non-infected) / Infected 2.0925 2.1734 96 1 0.711 0.4789

Status = Mated, Sex = Female, Time = 72:

contrast ratio SE df null t.ratio p.value

(Non-infected) / Infected 1.7726 1.8411 96 1 0.551 0.5828

Status = Mature, Sex = Female, Time = 72:

contrast ratio SE df null t.ratio p.value

(Non-infected) / Infected 0.0960 0.0998 96 1 -2.256 0.0264

Status = Virgin, Sex = Male, Time = 72:

contrast ratio SE df null t.ratio p.value

(Non-infected) / Infected 18.6139 19.3331 96 1 2.815 0.0059

Status = Mated, Sex = Male, Time = 72:

contrast ratio SE df null t.ratio p.value

(Non-infected) / Infected 0.9098 0.9450 96 1 -0.091 0.9277

Status = Mature, Sex = Male, Time = 72:

contrast ratio SE df null t.ratio p.value

(Non-infected) / Infected 0.8672 0.9007 96 1 -0.137 0.8912

Status = Virgin, Sex = Female, Time = 144:

contrast ratio SE df null t.ratio p.value

(Non-infected) / Infected 2.9470 3.0608 96 1 1.041 0.3007

Status = Mated, Sex = Female, Time = 144:

contrast ratio SE df null t.ratio p.value

(Non-infected) / Infected 2.2604 2.3477 96 1 0.785 0.4343

Status = Mature, Sex = Female, Time = 144:

contrast ratio SE df null t.ratio p.value

(Non-infected) / Infected 0.9960 1.0345 96 1 -0.004 0.9969

Status = Virgin, Sex = Male, Time = 144:

contrast ratio SE df null t.ratio p.value

(Non-infected) / Infected 0.1562 0.1622 96 1 -1.788 0.0770

Status = Mated, Sex = Male, Time = 144:

contrast ratio SE df null t.ratio p.value

(Non-infected) / Infected 1.6822 1.7472 96 1 0.501 0.6177

Status = Mature, Sex = Male, Time = 144:

contrast ratio SE df null t.ratio p.value

(Non-infected) / Infected 3.5585 3.6959 96 1 1.222 0.2247

Tests are performed on the log scale

**D - log(Defensin) ~ Status * Sex * Time * Infection**

**D.1 - Analysis of Variance Table**

Response: log(Defensin)

Df Sum Sq Mean Sq F value Pr(>F)

Status 2 11.459 5.7293 2.8590 0.06385 .

Sex 1 0.307 0.3069 0.1532 0.69669

Time 3 90.654 30.2179 15.0791 1.035e-07 ***

Infection 1 0.152 0.1521 0.0759 0.78371

Status:Sex 2 3.336 1.6678 0.8323 0.43921

Status:Time 6 21.344 3.5573 1.7751 0.11635

Sex:Time 3 0.538 0.1793 0.0895 0.96562

Status:Infection 2 5.131 2.5654 1.2802 0.28424

Sex:Infection 1 4.538 4.5382 2.2646 0.13673

Time:Infection 3 13.910 4.6366 2.3137 0.08312 .

Status:Sex:Time 6 17.683 2.9472 1.4707 0.20047

Status:Sex:Infection 2 2.872 1.4359 0.7165 0.49190

Status:Time:Infection 6 16.516 2.7527 1.3736 0.23693

Sex:Time:Infection 3 4.226 1.4086 0.7029 0.55340

Status:Sex:Time:Infection 5 23.956 4.7911 2.3908 0.04601 *

Residuals 72 144.285 2.0040

---

Signif. codes: 0 ‘***’ 0.001 ‘**’ 0.01 ‘*’ 0.05 ‘.’ 0.1 ‘ ’ 1

**D.2 – emmeans results**

Status = Virgin, Sex = Female, Time = 0:

contrast ratio SE df null t.ratio p.value

(Non-infected) / Infected 0.395 0.560 72 1 -0.656 0.5141

Status = Mated, Sex = Female, Time = 0:

contrast ratio SE df null t.ratio p.value

(Non-infected) / Infected 0.603 0.986 72 1 -0.309 0.7579

Status = Mature, Sex = Female, Time = 0:

contrast ratio SE df null t.ratio p.value

(Non-infected) / Infected 0.522 0.675 72 1 -0.503 0.6165

Status = Virgin, Sex = Male, Time = 0:

contrast ratio SE df null t.ratio p.value

(Non-infected) / Infected 1.323 2.294 72 1 0.162 0.8721

Status = Mated, Sex = Male, Time = 0:

contrast ratio SE df null t.ratio p.value

(Non-infected) / Infected 1.596 2.062 72 1 0.362 0.7186

Status = Mature, Sex = Male, Time = 0:

contrast ratio SE df null t.ratio p.value

(Non-infected) / Infected 0.261 0.338 72 1 -1.038 0.3026

Status = Virgin, Sex = Female, Time = 24:

contrast ratio SE df null t.ratio p.value

(Non-infected) / Infected 0.257 0.297 72 1 -1.175 0.2437

Status = Mated, Sex = Female, Time = 24:

contrast ratio SE df null t.ratio p.value

(Non-infected) / Infected 0.353 0.408 72 1 -0.901 0.3706

Status = Mature, Sex = Female, Time = 24:

contrast ratio SE df null t.ratio p.value

(Non-infected) / Infected 0.112 0.130 72 1 -1.893 0.0624

Status = Virgin, Sex = Male, Time = 24:

contrast ratio SE df null t.ratio p.value

(Non-infected) / Infected 36.822 63.841 72 1 2.080 0.0411

Status = Mated, Sex = Male, Time = 24:

contrast ratio SE df null t.ratio p.value

(Non-infected) / Infected 0.208 0.269 72 1 -1.215 0.2285

Status = Mature, Sex = Male, Time = 24:

contrast ratio SE df null t.ratio p.value

(Non-infected) / Infected 1.710 1.977 72 1 0.464 0.6439

Status = Virgin, Sex = Female, Time = 72:

contrast ratio SE df null t.ratio p.value

(Non-infected) / Infected 0.478 0.553 72 1 -0.638 0.5252

Status = Mated, Sex = Female, Time = 72:

contrast ratio SE df null t.ratio p.value

(Non-infected) / Infected 1.706 2.204 72 1 0.413 0.6807

Status = Mature, Sex = Female, Time = 72:

contrast ratio SE df null t.ratio p.value

(Non-infected) / Infected nonEst NA NA 1 NA NA

Status = Virgin, Sex = Male, Time = 72:

contrast ratio SE df null t.ratio p.value

(Non-infected) / Infected 2.290 2.647 72 1 0.717 0.4758

Status = Mated, Sex = Male, Time = 72:

contrast ratio SE df null t.ratio p.value

(Non-infected) / Infected 0.261 0.302 72 1 -1.161 0.2495

Status = Mature, Sex = Male, Time = 72:

contrast ratio SE df null t.ratio p.value

(Non-infected) / Infected 0.208 0.269 72 1 -1.214 0.2289

Status = Virgin, Sex = Female, Time = 144:

contrast ratio SE df null t.ratio p.value

(Non-infected) / Infected 15.161 26.285 72 1 1.568 0.1212

Status = Mated, Sex = Female, Time = 144:

contrast ratio SE df null t.ratio p.value

(Non-infected) / Infected 10.597 13.695 72 1 1.827 0.0719

Status = Mature, Sex = Female, Time = 144:

contrast ratio SE df null t.ratio p.value

(Non-infected) / Infected 0.263 0.339 72 1 -1.035 0.3042

Status = Virgin, Sex = Male, Time = 144:

contrast ratio SE df null t.ratio p.value

(Non-infected) / Infected 0.197 0.255 72 1 -1.257 0.2129

Status = Mated, Sex = Male, Time = 144:

contrast ratio SE df null t.ratio p.value

(Non-infected) / Infected 25.425 29.387 72 1 2.799 0.0066

Status = Mature, Sex = Male, Time = 144:

contrast ratio SE df null t.ratio p.value

(Non-infected) / Infected 4.518 5.222 72 1 1.305 0.1961

Tests are performed on the log scale

**E - log(Relish) ~ Status * Sex * Time * Infection**

**E.1 - Analysis of Variance Table**

Response: log(Relish)

Df Sum Sq Mean Sq F value Pr(>F)

Status 2 56.387 28.193 13.8778 5.157e-06 ***

Sex 1 5.940 5.940 2.9241 0.090533 .

Time 3 184.863 61.621 30.3321 7.656e-14 ***

Infection 1 3.166 3.166 1.5582 0.215000

Status:Sex 2 22.627 11.313 5.5689 0.005165 **

Status:Time 6 19.833 3.306 1.6271 0.148058

Sex:Time 3 4.941 1.647 0.8108 0.490974

Status:Infection 2 2.275 1.138 0.5600 0.573105

Sex:Infection 1 0.000 0.000 0.0001 0.993695

Time:Infection 3 6.017 2.006 0.9873 0.402238

Status:Sex:Time 6 8.285 1.381 0.6797 0.666337

Status:Sex:Infection 2 11.381 5.691 2.8011 0.065766 .

Status:Time:Infection 6 11.556 1.926 0.9480 0.464770

Sex:Time:Infection 3 3.014 1.005 0.4945 0.686968

Status:Sex:Time:Infection 6 10.397 1.733 0.8530 0.532495

Residuals 95 192.997 2.032

---

Signif. codes: 0 ‘***’ 0.001 ‘**’ 0.01 ‘*’ 0.05 ‘.’ 0.1 ‘ ’ 1

**E.2 – emmeans results**

Status = Virgin, Sex = Female, Time = 0:

contrast ratio SE df null t.ratio p.value

(Non-infected) / Infected 0.367 0.427 95 1 -0.862 0.3910

Status = Mated, Sex = Female, Time = 0:

contrast ratio SE df null t.ratio p.value

(Non-infected) / Infected 0.628 0.731 95 1 -0.400 0.6904

Status = Mature, Sex = Female, Time = 0:

contrast ratio SE df null t.ratio p.value

(Non-infected) / Infected 0.715 0.832 95 1 -0.289 0.7734

Status = Virgin, Sex = Male, Time = 0:

contrast ratio SE df null t.ratio p.value

(Non-infected) / Infected 0.831 0.967 95 1 -0.159 0.8740

Status = Mated, Sex = Male, Time = 0:

contrast ratio SE df null t.ratio p.value

(Non-infected) / Infected 0.230 0.268 95 1 -1.262 0.2101

Status = Mature, Sex = Male, Time = 0:

contrast ratio SE df null t.ratio p.value

(Non-infected) / Infected 0.361 0.420 95 1 -0.876 0.3835

Status = Virgin, Sex = Female, Time = 24:

contrast ratio SE df null t.ratio p.value

(Non-infected) / Infected 0.326 0.380 95 1 -0.963 0.3382

Status = Mated, Sex = Female, Time = 24:

contrast ratio SE df null t.ratio p.value

(Non-infected) / Infected 0.987 1.149 95 1 -0.011 0.9912

Status = Mature, Sex = Female, Time = 24:

contrast ratio SE df null t.ratio p.value

(Non-infected) / Infected 0.131 0.153 95 1 -1.743 0.0845

Status = Virgin, Sex = Male, Time = 24:

contrast ratio SE df null t.ratio p.value

(Non-infected) / Infected 12.087 15.727 95 1 1.915 0.0585

Status = Mated, Sex = Male, Time = 24:

contrast ratio SE df null t.ratio p.value

(Non-infected) / Infected 0.214 0.249 95 1 -1.325 0.1885

Status = Mature, Sex = Male, Time = 24:

contrast ratio SE df null t.ratio p.value

(Non-infected) / Infected 0.440 0.512 95 1 -0.705 0.4827

Status = Virgin, Sex = Female, Time = 72:

contrast ratio SE df null t.ratio p.value

(Non-infected) / Infected 0.576 0.670 95 1 -0.474 0.6363

Status = Mated, Sex = Female, Time = 72:

contrast ratio SE df null t.ratio p.value

(Non-infected) / Infected 8.067 9.388 95 1 1.794 0.0760

Status = Mature, Sex = Female, Time = 72:

contrast ratio SE df null t.ratio p.value

(Non-infected) / Infected 0.346 0.403 95 1 -0.912 0.3643

Status = Virgin, Sex = Male, Time = 72:

contrast ratio SE df null t.ratio p.value

(Non-infected) / Infected 0.241 0.280 95 1 -1.223 0.2243

Status = Mated, Sex = Male, Time = 72:

contrast ratio SE df null t.ratio p.value

(Non-infected) / Infected 0.965 1.124 95 1 -0.030 0.9760

Status = Mature, Sex = Male, Time = 72:

contrast ratio SE df null t.ratio p.value

(Non-infected) / Infected 1.734 2.018 95 1 0.473 0.6374

Status = Virgin, Sex = Female, Time = 144:

contrast ratio SE df null t.ratio p.value

(Non-infected) / Infected 1.393 1.622 95 1 0.285 0.7762

Status = Mated, Sex = Female, Time = 144:

contrast ratio SE df null t.ratio p.value

(Non-infected) / Infected 5.555 6.465 95 1 1.473 0.1440

Status = Mature, Sex = Female, Time = 144:

contrast ratio SE df null t.ratio p.value

(Non-infected) / Infected 0.396 0.461 95 1 -0.795 0.4286

Status = Virgin, Sex = Male, Time = 144:

contrast ratio SE df null t.ratio p.value

(Non-infected) / Infected 0.502 0.585 95 1 -0.591 0.5556

Status = Mated, Sex = Male, Time = 144:

contrast ratio SE df null t.ratio p.value

(Non-infected) / Infected 1.098 1.278 95 1 0.081 0.9360

Status = Mature, Sex = Male, Time = 144:

contrast ratio SE df null t.ratio p.value

(Non-infected) / Infected 3.102 3.610 95 1 0.973 0.3331

Tests are performed on the log scale

**F - log(PGRP.LC) ~ Status * Sex * Time * Infection**

**F.1 - Analysis of Variance Table**

Response: log(PGRP.LC)

Df Sum Sq Mean Sq F value Pr(>F)

Status 2 0.068 0.0341 0.0281 0.97226

Sex 1 0.466 0.4655 0.3847 0.53663

Time 3 4.203 1.4011 1.1579 0.33028

Infection 1 1.420 1.4199 1.1735 0.28155

Status:Sex 2 3.068 1.5339 1.2677 0.28641

Status:Time 6 11.493 1.9156 1.5831 0.16104

Sex:Time 3 11.908 3.9694 3.2804 0.02447 *

Status:Infection 2 10.856 5.4282 4.4860 0.01387 *

Sex:Infection 1 0.826 0.8257 0.6824 0.41093

Time:Infection 3 3.321 1.1071 0.9149 0.43703

Status:Sex:Time 6 4.749 0.7914 0.6541 0.68675

Status:Sex:Infection 2 0.807 0.4033 0.3333 0.71745

Status:Time:Infection 6 8.946 1.4910 1.2322 0.29727

Sex:Time:Infection 3 0.206 0.0686 0.0567 0.98215

Status:Sex:Time:Infection 6 3.764 0.6274 0.5185 0.79296

Residuals 91 110.113 1.2100

---

Signif. codes: 0 ‘***’ 0.001 ‘**’ 0.01 ‘*’ 0.05 ‘.’ 0.1 ‘ ’ 1

**F.2 – emmeans results**

Status = Virgin, Sex = Female, Time = 0:

contrast ratio SE df null t.ratio p.value

(Non-infected) / Infected 2.149 1.930 91 1 0.852 0.3967

Status = Mated, Sex = Female, Time = 0:

contrast ratio SE df null t.ratio p.value

(Non-infected) / Infected 1.636 1.643 91 1 0.490 0.6250

Status = Mature, Sex = Female, Time = 0:

contrast ratio SE df null t.ratio p.value

(Non-infected) / Infected 1.036 1.040 91 1 0.035 0.9722

Status = Virgin, Sex = Male, Time = 0:

contrast ratio SE df null t.ratio p.value

(Non-infected) / Infected 1.443 1.296 91 1 0.408 0.6840

Status = Mated, Sex = Male, Time = 0:

contrast ratio SE df null t.ratio p.value

(Non-infected) / Infected 0.918 0.825 91 1 -0.095 0.9243

Status = Mature, Sex = Male, Time = 0:

contrast ratio SE df null t.ratio p.value

(Non-infected) / Infected 0.798 0.717 91 1 -0.251 0.8025

Status = Virgin, Sex = Female, Time = 24:

contrast ratio SE df null t.ratio p.value

(Non-infected) / Infected 1.713 1.539 91 1 0.600 0.5503

Status = Mated, Sex = Female, Time = 24:

contrast ratio SE df null t.ratio p.value

(Non-infected) / Infected 1.869 1.679 91 1 0.697 0.4879

Status = Mature, Sex = Female, Time = 24:

contrast ratio SE df null t.ratio p.value

(Non-infected) / Infected 0.329 0.295 91 1 -1.238 0.2188

Status = Virgin, Sex = Male, Time = 24:

contrast ratio SE df null t.ratio p.value

(Non-infected) / Infected 1.473 1.479 91 1 0.386 0.7007

Status = Mated, Sex = Male, Time = 24:

contrast ratio SE df null t.ratio p.value

(Non-infected) / Infected 3.288 2.953 91 1 1.325 0.1884

Status = Mature, Sex = Male, Time = 24:

contrast ratio SE df null t.ratio p.value

(Non-infected) / Infected 0.141 0.141 91 1 -1.953 0.0539

Status = Virgin, Sex = Female, Time = 72:

contrast ratio SE df null t.ratio p.value

(Non-infected) / Infected 0.403 0.362 91 1 -1.011 0.3147

Status = Mated, Sex = Female, Time = 72:

contrast ratio SE df null t.ratio p.value

(Non-infected) / Infected 8.108 7.283 91 1 2.330 0.0220

Status = Mature, Sex = Female, Time = 72:

contrast ratio SE df null t.ratio p.value

(Non-infected) / Infected 0.271 0.273 91 1 -1.299 0.1974

Status = Virgin, Sex = Male, Time = 72:

contrast ratio SE df null t.ratio p.value

(Non-infected) / Infected 0.485 0.436 91 1 -0.805 0.4231

Status = Mated, Sex = Male, Time = 72:

contrast ratio SE df null t.ratio p.value

(Non-infected) / Infected 1.245 1.119 91 1 0.244 0.8075

Status = Mature, Sex = Male, Time = 72:

contrast ratio SE df null t.ratio p.value

(Non-infected) / Infected 0.880 0.790 91 1 -0.143 0.8869

Status = Virgin, Sex = Female, Time = 144:

contrast ratio SE df null t.ratio p.value

(Non-infected) / Infected 1.044 0.937 91 1 0.047 0.9623

Status = Mated, Sex = Female, Time = 144:

contrast ratio SE df null t.ratio p.value

(Non-infected) / Infected 7.427 6.670 91 1 2.232 0.0280

Status = Mature, Sex = Female, Time = 144:

contrast ratio SE df null t.ratio p.value

(Non-infected) / Infected 2.097 1.884 91 1 0.825 0.4117

Status = Virgin, Sex = Male, Time = 144:

contrast ratio SE df null t.ratio p.value

(Non-infected) / Infected 0.932 0.837 91 1 -0.078 0.9377

Status = Mated, Sex = Male, Time = 144:

contrast ratio SE df null t.ratio p.value

(Non-infected) / Infected 3.048 2.738 91 1 1.241 0.2178

Status = Mature, Sex = Male, Time = 144:

contrast ratio SE df null t.ratio p.value

(Non-infected) / Infected 1.327 1.192 91 1 0.315 0.7532

Tests are performed on the log scale

**G - log(Takeout) ~ Status * Sex * Time * Infection**

**G.1 - Analysis of Variance Table**

Response: log(Takeout)

Df Sum Sq Mean Sq F value Pr(>F)

Status 2 4.162 2.081 1.3914 0.253796

Sex 1 218.341 218.341 145.9725 < 2.2e-16 ***

Time 3 82.877 27.626 18.4693 1.681e-09 ***

Infection 1 2.261 2.261 1.5118 0.221935

Status:Sex 2 11.226 5.613 3.7525 0.027046 *

Status:Time 6 55.476 9.246 6.1814 1.702e-05 ***

Sex:Time 3 13.780 4.593 3.0709 0.031570 *

Status:Infection 2 14.939 7.470 4.9939 0.008686 **

Sex:Infection 1 8.011 8.011 5.3556 0.022834 *

Time:Infection 3 11.120 3.707 2.4781 0.066007 .

Status:Sex:Time 6 12.503 2.084 1.3931 0.225516

Status:Sex:Infection 2 9.704 4.852 3.2437 0.043429 *

Status:Time:Infection 6 12.237 2.040 1.3635 0.237432

Sex:Time:Infection 3 2.420 0.807 0.5393 0.656508

Status:Sex:Time:Infection 6 28.019 4.670 3.1220 0.007805 **

Residuals 94 140.602 1.496

---

Signif. codes: 0 ‘***’ 0.001 ‘**’ 0.01 ‘*’ 0.05 ‘.’ 0.1 ‘ ’ 1

**G.2 – emmeans results**

Status = Virgin, Sex = Female, Time = 0:

contrast ratio SE df null t.ratio p.value

(Non-infected) / Infected 0.5266 0.5258 94 1 -0.642 0.5223

Status = Mated, Sex = Female, Time = 0:

contrast ratio SE df null t.ratio p.value

(Non-infected) / Infected 0.6354 0.6345 94 1 -0.454 0.6508

Status = Mature, Sex = Female, Time = 0:

contrast ratio SE df null t.ratio p.value

(Non-infected) / Infected 0.6132 0.6124 94 1 -0.490 0.6255

Status = Virgin, Sex = Male, Time = 0:

contrast ratio SE df null t.ratio p.value

(Non-infected) / Infected 0.8298 0.8287 94 1 -0.187 0.8522

Status = Mated, Sex = Male, Time = 0:

contrast ratio SE df null t.ratio p.value

(Non-infected) / Infected 8.3460 8.3342 94 1 2.125 0.0362

Status = Mature, Sex = Male, Time = 0:

contrast ratio SE df null t.ratio p.value

(Non-infected) / Infected 0.0735 0.0734 94 1 -2.614 0.0104

Status = Virgin, Sex = Female, Time = 24:

contrast ratio SE df null t.ratio p.value

(Non-infected) / Infected 0.2591 0.2587 94 1 -1.353 0.1795

Status = Mated, Sex = Female, Time = 24:

contrast ratio SE df null t.ratio p.value

(Non-infected) / Infected 1.3623 1.3604 94 1 0.310 0.7575

Status = Mature, Sex = Female, Time = 24:

contrast ratio SE df null t.ratio p.value

(Non-infected) / Infected 0.3406 0.3402 94 1 -1.078 0.2836

Status = Virgin, Sex = Male, Time = 24:

contrast ratio SE df null t.ratio p.value

(Non-infected) / Infected 37.5425 37.4895 94 1 3.631 0.0005

Status = Mated, Sex = Male, Time = 24:

contrast ratio SE df null t.ratio p.value

(Non-infected) / Infected 0.1744 0.1947 94 1 -1.564 0.1211

Status = Mature, Sex = Male, Time = 24:

contrast ratio SE df null t.ratio p.value

(Non-infected) / Infected 2.4456 2.4422 94 1 0.896 0.3728

Status = Virgin, Sex = Female, Time = 72:

contrast ratio SE df null t.ratio p.value

(Non-infected) / Infected 0.7852 0.7841 94 1 -0.242 0.8092

Status = Mated, Sex = Female, Time = 72:

contrast ratio SE df null t.ratio p.value

(Non-infected) / Infected 2.5932 2.5896 94 1 0.954 0.3424

Status = Mature, Sex = Female, Time = 72:

contrast ratio SE df null t.ratio p.value

(Non-infected) / Infected 0.1318 0.1316 94 1 -2.029 0.0453

Status = Virgin, Sex = Male, Time = 72:

contrast ratio SE df null t.ratio p.value

(Non-infected) / Infected 3.3912 3.3865 94 1 1.223 0.2244

Status = Mated, Sex = Male, Time = 72:

contrast ratio SE df null t.ratio p.value

(Non-infected) / Infected 1.1690 1.1673 94 1 0.156 0.8761

Status = Mature, Sex = Male, Time = 72:

contrast ratio SE df null t.ratio p.value

(Non-infected) / Infected 1.3212 1.3193 94 1 0.279 0.7809

Status = Virgin, Sex = Female, Time = 144:

contrast ratio SE df null t.ratio p.value

(Non-infected) / Infected 1.3168 1.3149 94 1 0.276 0.7835

Status = Mated, Sex = Female, Time = 144:

contrast ratio SE df null t.ratio p.value

(Non-infected) / Infected 33.3417 33.2947 94 1 3.512 0.0007

Status = Mature, Sex = Female, Time = 144:

contrast ratio SE df null t.ratio p.value

(Non-infected) / Infected 0.2720 0.2716 94 1 -1.304 0.1954

Status = Virgin, Sex = Male, Time = 144:

contrast ratio SE df null t.ratio p.value

(Non-infected) / Infected 4.7461 5.2988 94 1 1.395 0.1663

Status = Mated, Sex = Male, Time = 144:

contrast ratio SE df null t.ratio p.value

(Non-infected) / Infected 4.5518 4.5453 94 1 1.518 0.1325

Status = Mature, Sex = Male, Time = 144:

contrast ratio SE df null t.ratio p.value

(Non-infected) / Infected 3.5165 3.5115 94 1 1.259 0.2111

Tests are performed on the log scale
